# Refractory Thrombocytopenia in TP53—Aberrant Chronic Lymphocytic Leukemia: A Multimechanistic Case with Response to Idelalisib and Romiplostim

**DOI:** 10.3390/jcm15124483

**Published:** 2026-06-10

**Authors:** Serkan Guven, Hulya Kacar, Omer Seker, Fatih Demirkan

**Affiliations:** 1 Department of Hematology, Canakkale Mehmet Akif Ersoy State Hospital, Ministry of Health, 17000 Canakkale, Turkey; 2 Department of Pathology, Canakkale Mehmet Akif Ersoy State Hospital, Ministry of Health, 17000 Canakkale, Turkey; hulyakacar.hk@gmail.com; 3 Department of Hematology, Sirnak State Hospital, Ministry of Health, 73000 Sirnak, Turkey; bensheker@hotmail.com; 4 Department of Hematology, Medstar Antalya Hospital, 07000 Antalya, Turkey; fatih.demirkan@yahoo.com

**Keywords:** bone marrow microenvironment, chronic lymphocytic leukemia, Del(17p), idelalisib, ımmune dysregulation, romiplostim, thrombocytopenia, TP53

## Abstract

**Background/Objectives:** Thrombocytopenia in chronic lymphocytic leukemia (CLL) is a heterogeneous and multifactorial complication that often reflects the combined effects of immune dysregulation, impaired megakaryopoiesis, bone marrow microenvironmental disruption, and disease-related factors. In patients with high-risk molecular features, particularly TP53 abnormalities, management becomes increasingly challenging and frequently refractory to conventional therapies. **Methods:** We report a 57-year-old male with long-standing CLL characterized by a highly aggressive and treatment-refractory course. The patient developed persistent severe thrombocytopenia despite multiple lines of therapy, including corticosteroids, intravenous immunoglobulin, rituximab, splenectomy, and thrombopoietin receptor agonists. Subsequent treatments with ibrutinib and a venetoclax-based regimen failed to improve platelet counts and were discontinued due to worsening cytopenia. Bone marrow evaluation, molecular/cytogenetic analyses, and subsequent treatment responses were thoroughly evaluated. **Results:** Bone marrow evaluation revealed hypercellularity with significant CLL infiltration, dysplastic megakaryopoiesis, and reticulin fibrosis, indicating impaired platelet production in addition to immune-mediated destruction. Molecular and cytogenetic analyses demonstrated high-risk disease with deletion of 17p and dual TP53 mutations (p.His179Tyr and p.Arg282Trp), consistent with biallelic TP53 disruption. Romiplostim monotherapy did not result in a meaningful hematologic response. However, following the addition of idelalisib, a rapid and sustained increase in platelet counts was observed, allowing tapering of romiplostim and stabilization of hematologic parameters. **Conclusions:** This case highlights the complex and dynamic pathophysiology of thrombocytopenia in CLL, where immune-mediated destruction and defective thrombopoiesis coexist within a profoundly altered marrow microenvironment. TP53 disruption appears to play a central role not only in driving treatment resistance but also in promoting immune dysregulation and disease aggressiveness. Although a delayed therapeutic effect of romiplostim cannot be entirely excluded, the distinct temporal association following idelalisib initiation suggests a potential collaborative interaction or disease-directed clearance that may facilitate platelet recovery in this setting. Refractory thrombocytopenia in CLL should be approached as a manifestation of complex disease biology rather than an isolated complication. This single observation indicates that in TP53 aberrant cases with multi-mechanism thrombocytopenia, disease-directed targeted therapy may contribute significantly to platelet recovery.

## 1. Introduction

Chronic lymphocytic leukemia (CLL) is characterized not only by clonal B cell proliferation but also by profound immune dysregulation involving both innate and adaptive immune compartments. This immune imbalance contributes to infectious susceptibility, impaired immune surveillance, and autoimmune complications, particularly autoimmune cytopenias such as immune thrombocytopenia (ITP). In contrast to primary ITP, thrombocytopenia in CLL is frequently multifactorial, involving both immüne-mediated platelet destruction and impaired platelet production related to marrow infiltration, microenvironmental disruption, and defective megakaryopoiesis. As a result, CLL-associated thrombocytopenia often demonstrates a more heterogeneous and treatment-refractory clinical course [1,2]. The clinical complexity becomes more pronounced in heavily pretreated patients, where progressive clonal evolution and acquisition of high-risk molecular abnormalities, particularly *TP53* disruption through del(17p) and/or *TP53* mutations, are associated with aggressive disease biology, treatment resistance, and poor clinical outcomes. Traditional approaches for CLL-associated thrombocytopenia, including corticosteroids, intravenous immunoglobulin, anti-CD20 therapies, splenectomy, and thrombopoietin receptor agonists, may be insufficient in refractory cases. Here, we present a heavily pretreated TP53-aberrant CLL patient with severe refractory thrombocytopenia in whom platelet recovery was observed following the combined use of romiplostim and idelalisib, illustrating that disease-directed targeted therapy can be a key component in managing cytopenias driven by complex disease biology [3,4].

## 2. Case Presentation

A 57-year-old male was diagnosed with chronic lymphocytic leukemia (CLL) in 2012. Peripheral blood flow cytometry at diagnosis demonstrated a typical CLL immunophenotype with CD5+, CD19+, CD23+, dim CD20 expression, and monoclonal light-chain restriction. Conventional cytogenetic and fluorescence in situ hybridization (FISH) analyses performed at diagnosis did not reveal any cytogenetic abnormalities. After presenting with lymphocytosis accompanied by significant cytopenias, most notably profound thrombocytopenia. From the outset, the presence of severe thrombocytopenia raised suspicion of an immune-mediated component, suggesting a complex disease biology beyond simple marrow infiltration. Due to the aggressive clinical presentation and the need for rapid disease control, treatment was initiated with R-CHOP chemoimmunotherapy.

Although an initial response was achieved, the disease course was characterized by early relapse and persistent hematologic instability. Over the following years, the patient required multiple lines of therapy, including fludarabine-based regimens and bendamustine-containing chemoimmunotherapy, reflecting a progressively refractory disease course and aggressive biological behavior.

During this period, thrombocytopenia evolved into the dominant and most challenging clinical problem. Platelet counts remained persistently low, with intermittent exacerbations requiring close monitoring and repeated therapeutic interventions. Standard treatments for immune thrombocytopenia, including prolonged corticosteroid therapy, intravenous immunoglobulin (IVIG), and rituximab, resulted only in transient and partial responses, supporting the presence of refractory CLL-associated immune thrombocytopenia.

Due to the lack of durable response, splenectomy was performed; however, platelet counts failed to normalize, indicating that splenic sequestration and destruction were not the sole mechanisms responsible for thrombocytopenia. This finding suggested a multifactorial pathogenesis involving both peripheral immune destruction and impaired platelet production.

Subsequently, thrombopoietin receptor agonist therapy with eltrombopag was initiated, yet the response remained suboptimal. This further supported the hypothesis that isolated stimulation of thrombopoiesis was insufficient in the presence of ongoing immune-mediated destruction and active leukemic disease. With continued disease activity, targeted therapy with ibrutinib was introduced and maintained for approximately four years. However, treatment was ultimately complicated by profound thrombocytopenia, necessitating discontinuation. Notably, despite severely reduced platelet levels, no major bleeding events occurred, likely due to meticulous clinical monitoring and supportive care. Following the discontinuation of ibrutinib due to severe thrombocytopenia, an additional targeted treatment approach was attempted using venetoclax in combination with eltrombopag. This strategy was selected with the aim of controlling the underlying disease and potentially improving the associated cytopenias. Therefore, despite the absence of active bleeding, this combination was considered in the context of persistently low platelet counts with the expectation that it might provide hematologic benefit. However, despite appropriate initiation and close monitoring, no improvement in platelet counts was observed. On the contrary, thrombocytopenia further worsened during this period. Due to the lack of hematologic response and progressive decline in platelet levels, the venetoclax plus eltrombopag combination was discontinued after approximately one month because of progressive worsening of thrombocytopenia and lack of hematologic response.

At this stage, the patient represented a highly complex clinical scenario characterized by heavily pretreated disease, refractory thrombocytopenia, and limited remaining therapeutic options.

**Figure 1:**
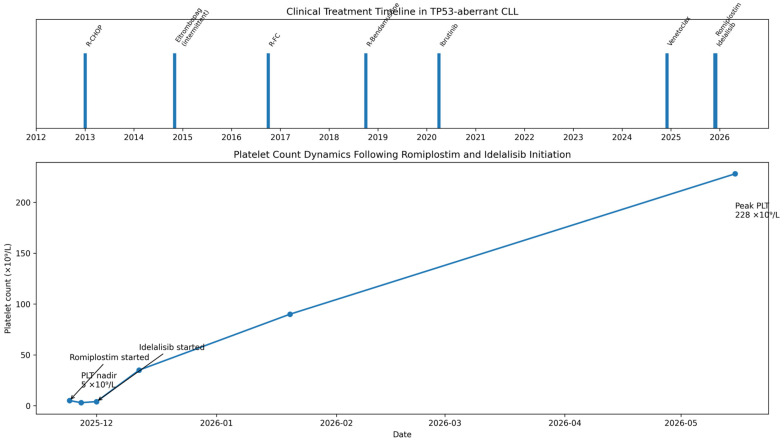
Clinical treatment timeline and schematic platelet count trend. The chart illustrates the sequential clinical treatment timeline (upper panel) and the corresponding longitudinal platelet count trend specifically for this single index patient (lower panel). Romiplostim was initiated as a salvage strategy during a critical thrombocytopenic nadir (5 × 10^9^/L), followed shortly thereafter by the introduction of idelalisib in December 2025 due to underlying disease progression, which preceded the subsequent robust and sustained elevation in platelet counts.

In November 2025, romiplostim was initiated with the aim of enhancing platelet production. Despite appropriate dosing and escalation, no clinically meaningful response was achieved, suggesting underlying impairment of megakaryocyte function and bone marrow microenvironmental disruption. A critical turning point occurred following a comprehensive reevaluation in 2025. Bone marrow biopsy and flow cytometric evaluation of the bone marrow confirmed persistent involvement by CLL with a characteristic immunophenotypic profile. Cytogenetic analysis demonstrated del(17p)/TP53 abnormality together with chromosome 12 gain (trisomy 12), supporting clonal evolution and high-risk disease biology. A repeat bone marrow biopsy performed in October 2025 revealed a hypercellular marrow (85–90% cellularity) featuring a nodular diffuse infiltration of chronic lymphocytic leukemia/small lymphocytic lymphoma (CLL/SLL) involving approximately 25–30% of the marrow space. Notably, megakaryocytic hyperplasia was observed (10–15 megakaryocytes/HPF), accompanied by prominent dysmorphic alterations, including micromegakaryocytes, pleomorphism, and multinucleated forms, alongside advanced reticulin fibrosis (Grade 2–3/4). Cytogenetic and molecular analyses demonstrated high-risk disease features. FISH revealed deletion of 17p13 involving the *TP53* locus in 65.5% of analyzed cells, and trisomy 12 was detected in 75% of cells. Next-generation sequencing (NGS) analysis further identified two pathogenic *TP53* mutations: Exon 5: c.535C>T (p.His179Tyr, VAF 45%) and Exon 8: c.844C>T (p.Arg282Trp, VAF 37%), strongly suggesting biallelic *TP53* disruption. Taken together, the etiology of thrombocytopenia in this patient was considered multifactorial, involving immune-mediated destruction, ineffective thrombopoiesis, bone marrow infiltration, and fibrosis-related microenvironmental impairment. Given the presence of TP53-aberrant disease and ongoing leukemic activity, idelalisib was subsequently initiated at a dose of 150 mg twice daily. Following its introduction, a rapid and sustained increase in platelet counts was observed (Figure 1). This hematologic improvement allowed gradual tapering of romiplostim while maintaining stable platelet levels. At the time of manuscript preparation, the patient remained on idelalisib therapy with stable platelet counts during approximately six months of follow-up, and no major idelalisib-related adverse events were observed.

## 3. Discussion

Thrombocytopenia in chronic lymphocytic leukemia (CLL) represents a biologically heterogeneous and clinically complex condition that rarely arises from a single dominant mechanism. Instead, particularly in heavily pretreated patients, it reflects the cumulative and dynamic interaction of immune dysregulation, impaired hematopoiesis, bone marrow microenvironmental disruption, and treatment-related effects. The present case exemplifies this complexity, illustrating how these mechanisms may coexist, evolve over time, and potentially contribute to a highly refractory clinical phenotype [5,6]. In this patient, the absence of a durable response to standard immunosuppressive strategies and splenectomy argues against a purely peripheral immune-mediated mechanism, while the lack of initial response to thrombopoietin receptor agonists indicates that production defects are simultaneously active. The profound immune dysregulation inherent to advanced CLL facilitates peripheral platelet destruction through autoreactive clones and altered T-cell milieus, creating a treatment-resistant loop [7]. Our bone marrow findings further clarify this production defect. The hypercellular marrow with dysplastic megakaryocytes, advanced reticulin fibrosis, and 25–30% nodular-diffuse leukemic infiltration demonstrates that ineffective thrombopoiesis must be understood within the broader context of a physically and functionally disrupted marrow ecosystem [8].

Leukemic infiltration itself represents an additional and often underappreciated contributor to cytopenia. In this case, 25–30% marrow involvement with a nodular diffuse pattern may have contributed to physical and functional displacement of normal hematopoietic elements. Thus, ineffective thrombopoiesis in this patient cannot be attributed solely to intrinsic megakaryocyte defects but must be understood within the broader context of a disrupted marrow ecosystem [9].

A defining feature of this case is the presence of high-risk molecular abnormalities involving the TP53 pathway. The coexistence of 17p deletion and pathogenic TP53 mutations with high variant allele frequencies is highly suggestive of biallelic inactivation. Biallelic TP53 disruption drives genomic instability and absolute treatment resistance. Beyond tumor cell proliferation, emerging data indicate that TP53 dysfunction exacerbates chronic immune activation and distorts apoptotic clearance, rendering associated cytopenias highly refractory to standalone conventional immunosuppressive or thrombopoietic therapies [10]. This multi-mechanism barrier explains why single-agent thrombopoietin receptor agonists or the combination of venetoclax and eltrombopag failed to restore hematopoietic balance in this advanced marrow environment. The introduction of idelalisib marked a clear turning point. As a selective inhibitor of the PI3Kδ pathway, idelalisib exerts dual effects, disrupting survival signaling in malignant B-cells while modulating T-cell signaling and reducing proinflammatory cytokine production [11,12].

The temporal relationship between treatment and response is highly informative. The rapid and sustained platelet recovery that occurred only after adding idelalisib to romiplostim highlights a clinical turning point. While a delayed therapeutic effect of romiplostim cannot be completely excluded, the distinct temporal alignment suggests that clone-directed therapy with idelalisib may have alleviated microenvironmental inhibition and immune-mediated peripheral clearance, thereby permitting romiplostim-stimulated megakaryopoiesis to manifest successfully [13].

Several limitations should be acknowledged. Mechanistic interpretations in this report remain hypothesis-generating, as serial immune profiling, platelet-associated antibody testing, thrombopoietin levels, immature platelet fraction analysis, and post-treatment marrow evaluation were not available. In addition, the relative contribution of delayed romiplostim response versus idelalisib-related immunomodulation cannot be definitively separated in a single-patient observation. 

In conclusion, this case illustrates the multifactorial nature of thrombocytopenia in TP53-aberrant CLL. The observed response to idelalisib indicates that targeted therapies capable of controlling the leukemic clone and modulating disease biology can provide vital hematologic benefit in selected refractory cytopenic states.

## Data Availability

Data supporting the findings of this study are available from the corresponding author upon reasonable request.

## References

[1] Vitale C., Ferrario A., Zamprogna G., Coscia M. (2025). Autoimmune Cytopenias in Chronic Lymphocytic Leukemia. Hematol. Oncol. Clin. N. Am..

[2] Fattizzo B., Barcellini W. (2020). Autoimmune Cytopenias in Chronic Lymphocytic Leukemia: Focus on Molecular Aspects. Front Oncol..

[3] Brieghel C., Kinalis S., Yde C.W., Schmidt A.Y., Jønson L., Andersen M.A., da Cunha-Bang C., Pedersen L.B., Geisler C.H., Nielsen F.C. (2019). Deep targeted sequencing of TP53 in chronic lymphocytic leukemia: Clinical impact at diagnosis and at time of treatment. Haematologica.

[4] Galitzia A., Maccaferri M., Mauro F.R., Murru R., Marasca R. (2024). Chronic Lymphocytic Leukemia: Management of Adverse Events in the Era of Targeted Agents. Cancers.

[5] Hallek M. (2025). Chronic Lymphocytic Leukemia: 2025 Update on the Epidemiology, Pathogenesis, Diagnosis, and Therapy. Am. J. Hematol..

[6] Gao P., Zhang Y., Ma J., Zhang Y. (2025). Immunotherapy in chronic lymphocytic leukemia: Advances and challenges. Exp. Hematol. Oncol..

[7] Oscier D., Dearden C., Eren E., Fegan C., Follows G., Hillmen P., Illidge T., Matutes E., Milligan D.W., Pettitt A. (2012). Guidelines on the diagnosis, investigation and management of chronic lymphocytic leukaemia. Br. J. Haematol..

[8] Vom Stein A.F., Hallek M., Nguyen P.H. (2024). Role of the tumor microenvironment in CLL pathogenesis. Semin. Hematol..

[9] Burger J.A., Gribben J.G. (2014). The microenvironment in chronic lymphocytic leukemia (CLL) and other B cell malignancies: Insight into disease biology and new targeted therapies. Semin. Cancer Biol..

[10] Blombery P., Chatzikonstantinou T., Gerousi M., Rosenquist R., Gaidano G., Pospisilova S., Roberts A.W., Birkinshaw R.W., Rossi D., Scarfo L. (2025). ERIC, the European Research Initiative on CLL. Resistance to targeted therapies in chronic lymphocytic leukemia: Current status and perspectives for clinical and diagnostic practice. Leukemia.

[11] Yavuz B., Karatas A.F., Erdogan Yucel E., Seker O., Guven S., Kakci M., Ozsan G.H., Alacacioglu I., Demirkan F. (2022). PB1889: A Case of Refractory Chronic Lymphocytic Leukemia Induced Immune Thrombocytopenic Purpura Successfully Treated with Venetoclax. HemaSphere.

[12] Ali K., Soond D.R., Pineiro R., Hagemann T., Pearce W., Lim E.L., Bouabe H., Scudamore C.L., Hancox T., Maecker H. (2014). Inactivation of PI(3)K p110δ breaks regulatory T-cell-mediated immune tolerance to cancer. Nature.

[13] Skånland S.S., Brown J.R. (2023). PI3K inhibitors in chronic lymphocytic leukemia: Where do we go from here?. Haematologica.

